# Management of hereditary angioedema with normal C1Inh: a series of 163 French patients

**DOI:** 10.1186/s13023-025-04155-8

**Published:** 2025-12-02

**Authors:** Alexis Bocquet, Laurence Bouillet, Gaelle Hardy, Isabelle Boccon-Gibod, Alban Deroux, Mélanie Arnaud, Fabien Pelletier, Aurélie Du Thanh, Nicolas Ozanne, Guillaume Armengol, Pierre Yves Jeandel, Laurent Sailler, Marie Caroline Taquet, David Launay, Olivier Fain, Delphine Gobert

**Affiliations:** 1 National Reference Center for Angioedema (CREAK), Angioedema Center of Reference and Excellence (ACARE), Grenoble Alpes University Hospital, Grenoble, France; 2 UM Génétique Moléculaire: Maladies Héréditaires et Oncologie, Institut de Biologie et Pathologie, CHU Grenoble Alpes, Grenoble, France; 3 Unité Transversale Allergologie, Dermatologie, CHU Besançon, Université de Franche-Comté, INSERM, UMR RIGHT, Besançon, France; 4 Department of Dermatology, University Hospital of Montpellier, Montpellier, France; 5 Service de Médecine Interne, CHU Charles Nicolle, Rouen, France; 6 Clinique Saint-Hilaire, Rouen, France; 7 Internal Medicine Department, Côte d’Azur University, CHU Nice, Hôpital l’Archet 1, Nice, France; 8 Internal Medicine Department, Hôpital Purpan, CHU Toulouse, Nice, France; 9 Internal Medicine Department, CHU de Strasbourg, Strasbourg, France; 10 CHU Lille, Service de Médecine Interne et Immunologie Clinique, CREAK, Lille, France; 11 U1286-INFINITE-Institute for Translational Research in Inflammation, Université de Lille, Lille, France; 12 Inserm, Lille, France; 13 Internal Medicine Department, CREAK, AP-HP, Hôpital Saint Antoine, 13- Sorbonne Université, Paris, France; 14 Centre National de Référence des Angioedèmes (CREAK), CHU Grenoble Alpes, CS 10217, Grenoble Cedex 09, 38043 France

**Keywords:** Angioedema, HAE-nC1INH, F12, Plasminogen

## Abstract

**Background:**

The diagnosis of hereditary angioedema with a normal C1Inh was genetic. The two most frequent pathogenic variants are found in *the* FXII and PLG genes. Their management is similar to that of HAE patients with C1Inh deficiency but without evidence-based medicine.

**Objective:**

The French Reference Centre for Angioedema (CREAK) Our center identified all patients with HAE with a normal C1Inh to evaluate their therapeutic management.

**Methods:**

This was a national retrospective study conducted in our center the CREAK network.

**Results:**

A total of 287 patients were identified with an *F12* pathogenic variant (133 families), 38 with *PLG* (12 families) and one patient with *KNG1*. Among these patients, 111 patients with HAE-FXII and 19 patients with HAE-PLG were symptomatic. More women than men were symptomatic (86.3% vs. 30.8%, respectively) (*p* < 0,0001). The mean age at first attack was 24 ± 12 years. 49% of patients with HAE-FXII were estrogen dependent (vs. 0% HAE-PLG, *p* < 0,01). 91% of patients with HAE-PLG needed to receive at least one attack of icatibant with 100% efficacy. 67% of patients with HAE-FXII were treated at least once: 56% with icatibant and 54% with C1Inh concentrate (during pregnancy). 12,6% of patients with HAE-FXII and 47,4% of patients with HAE-PLG required long-term prophylactic treatment: 66,7% of patients HAE-PLG who were taking tranexamic acid were attack-free (vs. 37,5 3% of HAE-FXII patients). 100% of patients with HAE-FXII treated with lanadelumab were completely asymptomatic (vs. 25% of patients with HAE-PLG).

**Conclusion:**

HAE patients with a normal C1inh have specific clinical features, including a later age at first attack than HAE patients with a normal C1inh, high sensitivity to estrogens of HAE-FXII and the location of the HAE-PLG on the tongue. The treatments used for HAE patients with C1Inh deficiency appear to be effective and safe. Low-dose progestin-only pills are good contraceptive options.

## Highlights

Hereditary angioedema (HAE) with normal C1Inh are very rare and seem to have different clinical characteristics to those of HAE with C1Inh deficiency.

Clinical differences exist according to the variant associated with HAE with normal C1inh, with slightly different therapeutic responses

It is important to determine the type of variant associated with HAE with normal C1Inh in order to tailor treatment to the patient’s needs.

## Introduction

Hereditary angioedema with normal C1Inh is a very rare disease that was identified for the first time in 2000 [1]. Since then, several pathogenic variants have been associated with this pathology. The main ones are *F12* p.(Thr328Lys) and *PLG* p.(Lys330Glu), described in several families and in several countries [2–7]. Other rarer pathogenic variants have been reported: *KNG1*,* ANGPT1*,* MYOF*,* and HS3ST6* [8–11]. The clinical symptoms are comparable to those of C1Inh deficiency in terms of the duration and location of attacks. The differences with HAE due to C1inh deficiency rely on the sensitivity to estrogens of these forms [2, 12], especially those related to *F12* pathogenic variants: almost all symptomatic patients are women, and female hormonal events (pregnancy, contraception, menstruations, etc.) are more frequently a trigger for angioedema attacks [2–5].

There are many pathophysiological arguments to suggest that HAE-FXII and HAE-PLG are mediated by bradykinin via dysfunction of the kallikrein-kinin pathway: *F12* pathogenic variants induce new sites that are sensitive to enzymatic cleavage by plasmin [13]. Therefore, FXII is rapidly activated after cleavage by plasmin, escapes from inhibition through a C1 inhibitor (C1Inh), and elicits excessive bradykinin formation. A gain-of-function pathogenic variant of PLG results in increased bradykinin release via direct cleavage of high-molecular-weight kininogen (HMWK) [14]. The *PLG* pathogenic variant bypasses FXII/kallikrein to generate bradykinin. Without evidence-based medicine but with these physiopathological arguments, we used the following HAE-C1Inh treatments: B2 receptor antagonist, anti-Kallikrein. However, it is important to evaluate these treatments in terms of both efficacy and safety. We therefore decided to analyze the therapeutic management of all French our patients.

## Patients and methods

This is a national, multicenter, retrospective study carried out by the CREAK our network (www.creakfr.org). Each patient identified with an *F12* and *PLG* pathogenic variant at our Grenoble University Hospital Molecular Biology Laboratory was included. Pathogenic variants were identified using targeted Sanger sequencing. All patients signed a consent form for the genetic study to be carried out. We have followed the reference methodology for the processing of personal data in the context of research not involving the human person (MR-004): Written information was given to patients when their information was entered into the HAE with normal C1INH study. Oral consent was obtained. A patient is labelled symptomatic if they have had at least one attack in their lifetime. The treatment of attack was considered effective by the specialist if the attack duration was reduced by at least 50% compared to the duration of untreated attacks. Statistical analysis: nonparametric Fischer and Wilcoxon-Mann‒Whitney tests.

## Results

### Population description

**Fig. 1:**
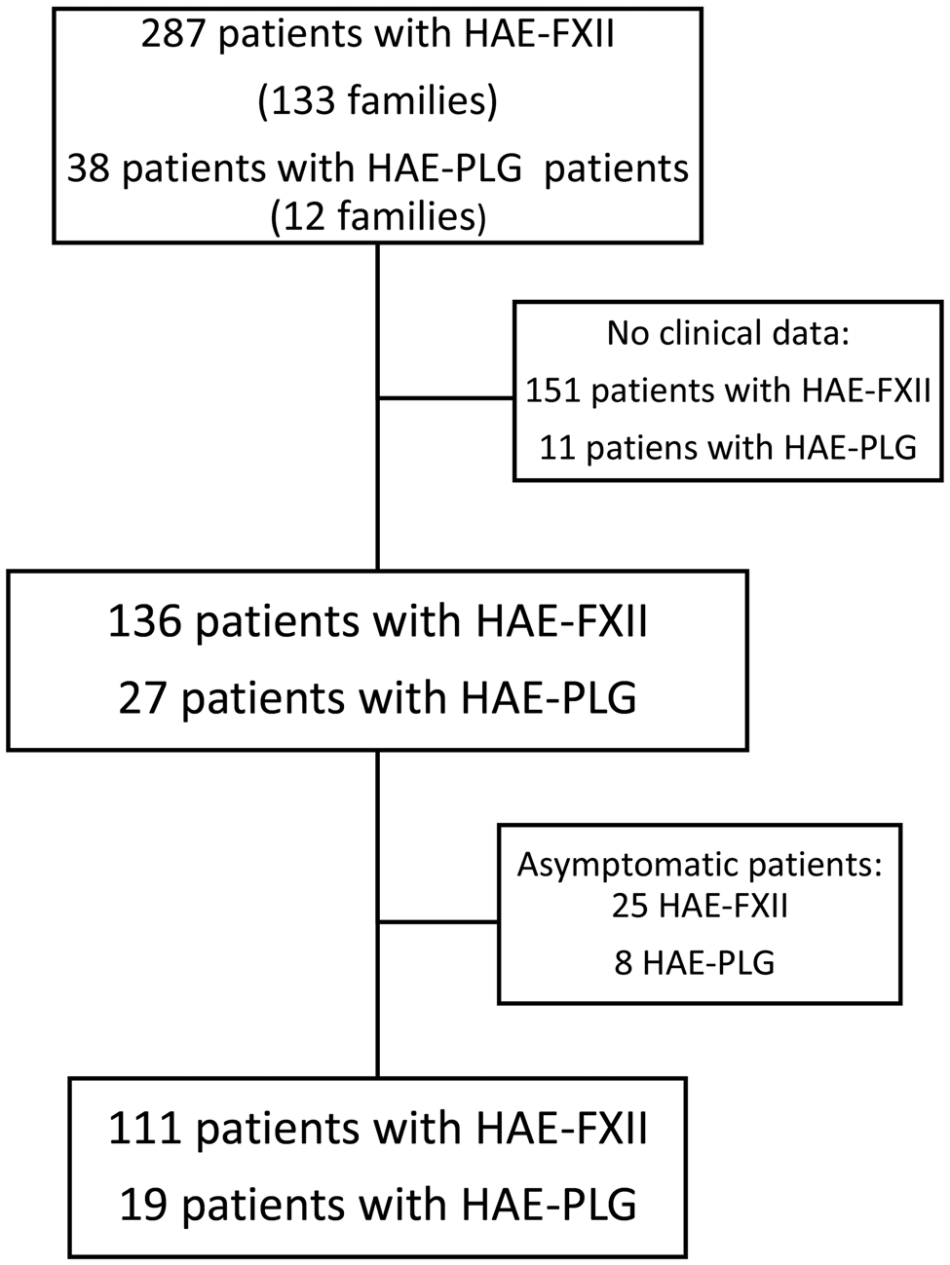
Flow chart

Two hundred eighty-seven patients were identified with an *F12* pathogenic variant (133 families), thirty-eight with a *PLG* pathogenic variant (12 families) and one with a KNG1 probable pathogenic variant. Clinical data were recovered from 136 patients with HAE-FXII and 27 patients with HAE-PLG. Among these patients, 111 patients with HAE-FXII (81.6%) and 19 patients with HAE-PLG (70.4%) were symptomatic (Fig. 1).

93,1% of patients HAE-PLG and 78,3% of patients HAE-FXII reported at least one family history of angioedema. 3,86% and 4% respectively reported at least one death from angioedema in their family.

We present data only for symptomatic patients. More women than men are symptomatic:

92% vs. 31% (*p* < 0, 0001). The median ages at the first attack were 24.5 and 24 years, respectively (Tables 1 and 2). 69% of patients with HAE-FXII were from North Africa, but none of these patients had HAE-PLG (*p* < 0, 0001). 86% of patients with HAE-FXII and 74% of patients with HAE-PLG had at least one facial attack; 68.5% of patients with HAE-PLG had at least one episode of angioedema of the tongue, whereas only 11% of patients with HAE-FXII did (*p* < 0, 0001). 45% of patients with HAE-FXII (and 31.5% of patients with HAE-PLG) had at least a laryngeal attack, and 67% had an abdominal attack (and 56% of patients with HAE-PLG).

**Table 1:** Description of patients with HAE-FXII patients according to country

	France	Italy (16)		Germany (2)		Spain (17,5)				Brazil (3)	
Symptomatic patients with HAE-FXII patients	111	20	p-value*	72	p-value*	22	p-value*	29	p-value*	102	p-value*
Sexe: Female/male	105/6	16/4	**0.046**	71/1	0.07	20/2	NS	26/3	NS	86/16	**0.022**
Age of first attack (mean)	24.5 years	20.5 years	NS	20.3 years	NS	20 years	NS	23.9 years	NS	21.1 years	NS
Attack’s localization Face Laryngeal Tongue Abdominal	86% 45% 11% 67%	85.7% 42.9% 28.5% 85%	NS NS **0.033** 0.12	100% 24,6% 32.8% 65.6%	**< 0.001** **0**,**008** **< 0.001** NS	75% 87.5% 43.8% 87.5%	NS **< 0.001** **< 0.001** 0.078	NA 17% NA 34%	**0.009** **0.002**	55.5% 32.3% 12.1% 69,7%	**< 0.0001** 0.068 NS NS
Estrogen dependent	49%	0%	**< 0.0001**	NA		43.8%	NS	96%	**< 0.0001**	72.8%	**< 0.001**
Triggering/aggravating factors^a^ Estrogen containing pills (ECP) Pregnancies ACEi	96.7% 52% 1	100% 78% 2	NS 0.11 0.06	90.7% 40% 3	0.092 0.12 NS	92.3% 61% 1	0.18 NS NS	NA NA 1	NS	83% 20% 1	**< 0.001** **< 0.0001** NS

A: percentage calculated from the total number of pregnancies occurrence and ECP use. NA: not available.*The p-value is determined by comparison with patients in our study (univariate analysis). Any p-value greater than 0.2 is noted as NS

**Table 2:** Description of patients with HAE-PLG according to country

	France	Germany (6)	
Symptomatic patient with HAE-PLG	19	60	p-value*
Sexe: Female/male	17/2	47/13	NS
Age of first attack	24 years	30,5 years	NS
Attack’s localization Face Laryngeal Tongue Abdominal	74% 31.5% 68.5% 56%	76,7% 10% 78.3% 30%	NS **0.033** NS 0.054
Estrogen dependent	0%	NA	
Triggering/aggravating factors^a^ Estrogen containing pills Pregnancies ACEi	100% (5 patients) 50% (3 pregnancies) 100% (4 patients)	14% (43 patients) 0% (73 pregnancies) NA	**< 0.001** NS

NA: not available. A: percentage calculated from the total number of pregnancies occurrence and ECP use. *The p-value is determined by comparison with patients in our study (univariate analysis). Any p-value greater than 0.2 is noted as NS

49% of patients with HAE-FXII were estrogen dependent, i.e., they only had attacks during pregnancy and/or when taking estrogen producers. However, none of the patients with HAE-PLs were affected (*p* < 0, 01). The contraceptive pill was a revealing or aggravating factor in 64% of patients with HAE-FXII and in 10% of patients with HAE-PLG. Angiotensin converting enzyme inhibitors (ACEis) were revealing factors in 21% of patients with HAE-PLG (none of whom had HAE-FXII). For one patient with HAE-FXII, her illness worsened under treatment with ACEis.

The mean duration of attacks was 60 ± 25 h for patients with AEH-FXII and 48 ± 24 h for patients with HAE-PLG. The mean frequency of attacks over the last 12 months was 0.71 ± 25 2.31/month for patients with HAE-FXII and 0.85 ± 1.50/month for patients with HAE-PLG. The mean maximum frequency of attacks was 1.39 ± 1.72/month for patients with HAE-FXII and 1.45-± 1.43/month for patients with HAE-PLG.

### On-demand treatment

91% of patients with HAE-PLG used on-demand treatment at least once, and 90% used icatibant. 67% of patients with HAE-FXII have received on-demand treatment: 56% of them have received icatibant, and 54% have received C1Inh concentrate (mainly during pregnancy). Icatibant and C1inh concentrate were effective for 98% of patients. No safety alerts were reported.

### Long-term prophylaxis

Due to frequent attacks, 47,4% patients with HAE-PLG (9 patients) and 12,6% patients with HAE-FXII (14 patients) required long-term prophylaxis. 87% were women. Seven patients with HAE-FXII and six patients with HAE-PLG patients have tried tranexamic acid (TA): 66,7% patients with HAE-PLG were attack-free vs. 37,5% patients with HAE-FXII. Five HAE-FXII and four HAE-PLG patients were given anti kallikrein drugs: 100% patients with HAE-FXII were completely asymptomatic vs. 25% patients with HAE-PLG (Table 3). Four women with HAE-FXII were treated with C1inh concentrate during pregnancy. No safety alert was reported.

**Table 3:** Long-term prophylaxis efficacy

	HAE-FXII	HAE-PLG
Patients requiring LTP	14 /111 (12,6%)	9/19 (47,4%)
Attack free with tranexamic acid	3/8 (37,5%)	4/6 (66,7%)
Attack free with lanadelumab	5/5 (100%)	1/3 (33%)
Attack free with berotralsat	None	0/1

### Contraception

31% of women with HAEH-FXII and 41% of women with HAE-PLG were taking contraception. Desogestrel was taken by 55% of women with HAE-FXII and 29% of women with HAE-PLG. Chlormadinone was used by 17% of women with HAE-FXII and 57% of women with HAE-PLG. These contraceptives are well tolerated without aggravating the disease.

### HAE-KNG1

The only patient with a kininogen pathogenic variant was a 50-year-old woman with a history of recurrent facial swelling, including of the lips and tongue, lasting 3 to 4 days. The use of icatibant for on-demand treatment, as well as TA for long-term prophylaxis, is effective (annual attack rate has been reduced by 100%).

## Discussion

We describe here one of the largest HAE patients with a normal C1Inh series; only Brazil and Germany have published comparable series [2, 3]. There are probably several explanations for this: these three countries may share clusters of populations, and screening and access to molecular diagnosis may be easier in these countries (France, Germany, and Brazil). We identified a particular cluster for patients with HAE-FXII: 69% of them were from North Africa. Neither our German nor Brazilian colleagues reported this particularity. This special feature prompted us to question our colleagues in the Maghreb and to envisage genetic studies with them to determine the prevalence of this disease in their populations.

It is interesting to note that the age of onset of symptoms is later than that of HAE patients with a normal C1Inh. Thus, the mean age at first attack was 14 years in our patients with HAE-C1Inh, i.e., 10 years earlier than in patients with HAE with normal C1Inh [15]. This later age for HAE patients with normal C1Inh is probably explained by their high estrogen sensitivity. The use of a pill or pregnancy often triggers the first attack.

The impact of estrogens (exogenous and endogenous) has been confirmed for patients with HAE-FXII. Approximately 83% to 100% of women with HAE-FXII who are taking an estrogen-containing pill (ECP) experience a worsening of their disease (Table 1) [2, 3, 5, 16, 17]. A total of 20.3% to 77.8% of women with HAE-FXII reported that their pregnancy worsened their illness [2, 3, 5, 16, 17]. Thus, 43.8% to 96% of women with HAE-FXII are estrogen dependent, i.e., they do not have any attacks apart from pregnancy or ECP (Table 1). This explains why, in the end, only 11.7% of women with HAE-FXII in our series needed long-term prophylaxis versus 59.2% of patients with HAE-C1Inh [18]. HAE-PLG is a very rare form. It seems to have one particular feature: frequent localization of attacks on the tongue. This is reported by 78.3% of German patients and 68.5% of French patients [6], whereas it is reported by only 11% to 43.8% (depending on the series published) of patients with HAE-FXII [2, 3, 5, 16, 17] (Table 2). ACE inhibitors also appear to be a revealing or aggravating factor in patients with HAE-FXII and HAE-PLG. ACE inhibitors are known to promote bradykinin-mediated AE by blocking the degradation of bradykinin by the converting enzyme [19]. It has been estimated that 0.1% to 0.7% of patients taking ACE inhibitors develop bradykinin-mediated AE [20]. The majority likely do not have a *PLG* or *F12* pathogenic variant. Systematic screening for this pathogenic variant should probably not be carried out in patients who develop BK-AE on ACE inhibitors unless the patient reports a family history of angioedema [21].

Treatment of attacks with icatibant is reported to be effective by 98% of patients. We had already demonstrated this efficacy thanks to the IOS register in 2017 [22]: the median time to resolution of attack in the HAE-C1INH group was 20.0 h. Icatibant was self-administered for 96.1% of attacks. No serious adverse side effects related to icatibant were reported. In our series, plasma-derived C1inh concentrate was only used as a treatment during pregnancy and had good efficacy. K. Bork reported data on the efficacy of C1inh concentrate on 143 facial attacks; compared with 88 untreated attacks, the duration of AEs was significantly reduced: 25.6 h instead of 64.1 h [2].

TA seems to be effective as a long-term prophylaxis in these conditions, especially in patients with HAE-PLG. This has also been reported by other authors ( [2, 3, 5, 16], and [17]). K. Bork reported a 93.8% reduction in the frequency of attacks in four women with HAE-FXII [2]. As we had already shown in our study published in 2014, TA is more effective for HAE patients with normal C1Inh than for those with C1Inh deficiency [23].

Antikallicrein agents (berotralstat and lanadelumab) are new and effective treatments for patients with HAE-C1Inh with few side effects. In 2021, we published the first two patients with HAE-FXII who were treated with lanadelumab [24]. Our series reports five more patients, i.e., seven patients in total. 100% of patients with HAE-FXII treated with anti-kalikrein drugs were completely asymptomatic, whereas 67% of patients with HAE-PLG were asymptomatic. This difference in efficacy may be explained by the fact that the *PLG* mutant bypasses FXII/kallikrein to generate bradykinin [14]. However, our sample size is small, and no conclusions can be drawn at this stage.

The subject of contraception is critical for HAE: women are the most symptomatic, and estrogens aggravate the pathology. Therefore, ECP is contraindicated for women. It is recommended to prescribe progestin only pills. In 2012, we published a study showing that antigonadotropic progestins (AGP) could be used not only as a contraception but also as a highly effective disease-modifying treatment [25]. Total or partial improvement was observed in 89.5% of patients taking AGP, compared to 61.3% of patients taking low-dose progestin only pills (*p* = 0.013). Unfortunately, in 2020, our medical agency issued a warning about AGP because of an increased risk of meningioma. For women treated for more than 6 months with AGP, the risk increased by 3.3 times compared with the baseline risk, 12.5 times from a cumulative dose corresponding to 5 years of treatment for nomgestrol acetate, and 7 times from a cumulative dose corresponding to 3.5 years of use of chlormadinone acetate [26]. AGP was gradually discontinued; in our study, only nine patients were still taking chlormadinone acetate. Low-dose progestin-only pills (desogestrel) do not present this risk: they are well tolerated as contraception and do not worsen the disease. Bork even reported that 93.7% of patients on desogestrel are asymptomatic [2].

Our study has limitations due to the retrospective nature of the data. Unfortunately, in the case of very rare diseases, this is often the only way of obtaining clinical and therapeutic data. These studies are important for assessing the safety of off-label use of these drugs. There have been no reports of side effects.

In conclusion, HAE patients with a normal C1inh have specific clinical features, including a later age at first attack than HAE patients with C1inh deficiency, high sensitivity to estrogens of HAE-FXII and the location of the HAE-PLG on the tongue. The treatments used for HAE patients with C1Inh deficiency appear to be effective and safe. Low-dose progestin-only pills are good contraceptive options. Hereditary angioedema with normal C1Inh are very rare and seem to have different clinical characteristics to those of HAE with C1Inh deficiency. Clinical differences exist according to the variant associated with HAE with normal C1inh, with slightly different therapeutic responses. Future challenges will be to determine the best treatments for each category of HAE with normal C1Inh.

## Data Availability

The datasets generated and/or analysed during the current study are not publicly available due to french data protection regulations but are available from the corresponding author on reasonable request.

## Abbreviations

AE: Angioedema
HAE: Hereditary angioedema
HAE-nC1INH: Hereditary angioedema with normal C1Inh
HAE-C1Inh: Hereditary angioedema with C1Inh deficiency
PLG: Plasminogen
KNG: Kininogen
C1Inh: C1 inhibitor
HMWK: High-molecular-weight kininogen
ACEi: Angiotensin converting enzyme inhibitors
TA: Tranexamic acid
AGP: Antigonadotropic progestin
ECP: Estrogen-containing pills
